# Lithium diaqua­nickel(II) *catena*-borodiphosphate(V) monohydrate

**DOI:** 10.1107/S1600536809014652

**Published:** 2009-05-07

**Authors:** Juan Zheng, Aiyun Zhang

**Affiliations:** a Department of Physics and Chemistry, Henan Polytechnic University, Jiaozuo 454000, People’s Republic of China

## Abstract

The title borophosphate LiNi(H_2_O)_2_[BP_2_O_8_]·H_2_O was synthesized under hydro­thermal conditions. The crystal structure is isotypic with the Mg analogue and features helical [BP_2_O_8_]^3−^ borophosphate ribbons, constructed by BO_4_ (2 symmetry) and PO_4_ tetra­hedra. The borate groups share all their oxygen apices with adjacent phosphate tetra­hedra. The ribbons are connected *via* Ni^2+^ cations that are located on twofold rotation axes. The cations have a slightly distorted octa­hedral oxygen coordination by four O atoms from the anion and by two water mol­ecules. The voids within the helices are occupied by Li^+^ cations, likewise located on twofold rotation axes, in an irregular environment of five O atoms. The structure is stabilized by O—H⋯O hydrogen bonds between coordinated or uncoordinated water mol­ecules and O atoms that are part of the helices.

## Related literature

For the isotypic Mg analogue, see: Lin *et al.* (2008 ▶). For other borophosphates, see: Boy & Kniep (2001 ▶); Kniep *et al.* (1998 ▶). A review on the structural chemistry of borophosphates is given by Ewald *et al.* (2007 ▶).

## Experimental

### 

#### Crystal data

LiNi(H_2_O)_2_[BP_2_O_8_]·H_2_O
*M* *_r_* = 320.44
Hexagonal, 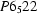

*a* = 9.3359 (3) Å
*c* = 15.7497 (11) Å
*V* = 1188.82 (10) Å^3^
*Z* = 6
Mo *K*α radiation
μ = 2.91 mm^−1^
*T* = 296 K
0.22 × 0.20 × 0.17 mm

#### Data collection

Bruker APEXII CCD area-detector diffractometer
Absorption correction: multi-scan (*SADABS*; Bruker, 2007 ▶) *T*_min_ = 0.567, *T*_max_ = 0.638
6139 measured reflections
708 independent reflections
684 reflections with *I* > 2σ(*I*)
*R*_int_ = 0.049

#### Refinement

*R*[*F* ^2^ > 2σ(*F* ^2^)] = 0.026
*wR*(*F* ^2^) = 0.065
*S* = 1.14
708 reflections
75 parameters
H-atom parameters constrained
Δρ_max_ = 0.68 e Å^−3^
Δρ_min_ = −0.39 e Å^−3^
Absolute structure: Flack (1983 ▶), 235 Friedel pairs
Flack parameter: 0.01 (3)

### 

Data collection: *APEX2* (Bruker, 2007 ▶); cell refinement: *SAINT* (Bruker, 2007 ▶); data reduction: *SAINT*; program(s) used to solve structure: *SHELXS97* (Sheldrick, 2008 ▶); program(s) used to refine structure: *SHELXL97* (Sheldrick, 2008 ▶); molecular graphics: *SHELXTL* (Sheldrick, 2008 ▶); software used to prepare material for publication: *SHELXTL*.

## Supplementary Material

Crystal structure: contains datablocks I, global. DOI: 10.1107/S1600536809014652/wm2227sup1.cif

Structure factors: contains datablocks I. DOI: 10.1107/S1600536809014652/wm2227Isup2.hkl

Additional supplementary materials:  crystallographic information; 3D view; checkCIF report

## Figures and Tables

**Table 1:** Selected bond lengths (Å)

Ni1—O1^i^	2.048 (3)
Ni1—O2	2.070 (3)
Ni1—O3	2.130 (3)
P2—O1	1.503 (3)
P2—O2	1.510 (3)
P2—O4	1.546 (3)
P2—O5	1.556 (3)
O6—Li	2.12 (2)
B—O5^ii^	1.461 (5)
B—O4^iii^	1.471 (5)
Li—O2^iv^	2.113 (13)
Li—O3^v^	2.164 (4)

Symmetry codes: (i) 
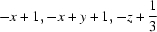
; (ii) 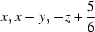; (iii) 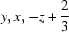; (iv) 
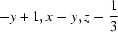
; (v) 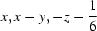.

**Table 2:** Hydrogen-bond geometry (Å, °)

*D*—H⋯*A*	*D*—H	H⋯*A*	*D*⋯*A*	*D*—H⋯*A*
O3—H3*A*⋯O5^iv^	0.81	2.01	2.746 (4)	151
O3—H3*A*⋯O2^iv^	0.81	2.60	3.165 (4)	128
O6—H6⋯O4^vi^	0.83	2.52	3.331 (4)	167
O6—H6⋯O1^vi^	0.83	2.66	3.092 (4)	114
O3—H3*B*⋯O1	0.83	2.00	2.810 (4)	167
O3—H3*B*⋯O2	0.83	2.54	2.955 (4)	112

Symmetry codes: (iv) 
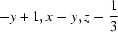
; (vi) 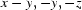.

**Fig. 1.**
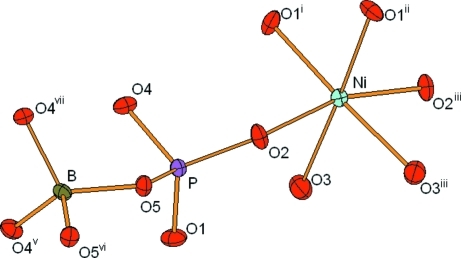
A part of the structure of LiNi(H2O)2[BP2O8].H2O with displacement ellipsoids drawn at the 50% the probability level. Symmetry codes: (i) 1 - y, 1 - x, 0.16667 - z; (ii) 1 - x, 1 - x + y, 0.33333 - z; (iii) x-y, x, -0.16667 + z; (iv) y, x, 0.66667 - z; (v) y, -x + y, 0.16667 + z; (vi) x, x-y, 0.83333 - z.

**Fig. 2.**
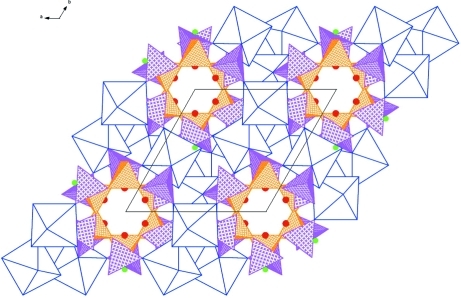
Polyhedral diagram for LiNi(H2O)2[BP2O8].H2O in projection along [001]. Colour code: purple P, orange B, blue Ni, red OW6 and green Li.

## supplementary crystallographic information

### Comment

With increasing interest in microporous materials, the synthesis of compounds like borophosphates with open framework structures have drawn much attention during the past few years. These compounds show a rich crystal chemistry (Kniep *et al.*, 1998; Ewald *et al.*, 2007).

The crystal structure of LiNi(H_2_O)_2_[BP_2_O_8_]_^.^_H_2_O is isotypic with that of the Mg analogue (Lin *et al.* 2008) and contains an infinite one-dimensional anionic structure. The condensation of BO_4_ and PO_4_ tetrahedra leads to helical ribbons with composition [BP_2_O_8_]^3-^ (Fig. 1), whereby each BO_4_ tetrahedron shares its vertices with four PO_4_ tetrahedra. Bond lenghts and angles within the anionic structure are consistent with related borophosphates (Boy & Kniep, 2001; Lin *et al.*, 2008).

The free loop of the borophosphate helix is occupied by Li^+^ cations, which are coordinated by with five O atoms, two from phosphate groups (O2) and three from water molecules (O3), thus completing an helical unit {Li[BP_2_O_8_]^2-^} with a central channel running along the 6_5_ screw axis. The channels are filled up with water of crystallization (O6). The Ni^2+^ cations, located on a twofold rotation axis, are surrounded in a distorted octahedral coordination by four O atoms from adjacent phosphate groups and two water molecules, leading to the overall formula LiNi(H_2_O)_2_[BP_2_O_8_]_^.^_H_2_O  (Fig. 2). The Ni—O distances range from 2.048 (3)–2.130 (3) Å and are in the usual range. The crystal structure is stabilized by O—H···O hydrogen bonds between coordinated or uncoordinated water molecules and O atoms that are part of the helices.

### Experimental

Green block-shaped crystals were synthesized hydrothermally from a mixture of Ni(NO_3_)_2_, Li_2_B_4_O_7_, water and H_3_PO_4_. In a typical synthesis, 0.87 g Ni(NO_3_)_2^.^_6H_2_O was dissolved in a mixture of 5 mL water, 1.691 g Li_2_B_4_O_7_ and 2 ml H_3_PO_4_ (85%_wt_ ) under constant stirring. Finally, the mixture was kept in a 30 ml Teflon-lined steel autoclave at 443 K for 6d. The autoclave was slowly cooled to room temperature.

### Refinement

The highest peak in the difference map is 1.29Å from atom H6, and the minimum peak is 0.48Å from atom P2.

### Crystal data

**Table:**

LiNi(H_2_O)_2_[BP_2_O_8_]·H_2_O	*D*_x_ = 2.686 Mg m^−^^3^
*M**_r_* = 320.44	Mo *K*α radiation, λ = 0.71073 Å
Hexagonal, *P*6_5_22	Cell parameters from 1684 reflections
Hall symbol: P 65 2 ( 0	θ = 2.5–29.5°
*a* = 9.3359 (3) Å	µ = 2.91 mm^−^^1^
*c* = 15.7497 (11) Å	*T* = 296 K
*V* = 1188.82 (10) Å^3^	Block, green
*Z* = 6	0.22 × 0.20 × 0.17 mm
*F*(000) = 960	

### Data collection

**Table:**

Bruker APEXII CCD area-detector diffractometer	708 independent reflections
Radiation source: fine-focus sealed tube	684 reflections with *I* > 2σ(*I*)
graphite	*R*_int_ = 0.049
φ and ω scans	θ_max_ = 29.5°, θ_min_ = 2.5°
Absorption correction: multi-scan (*SADABS*; Bruker, 2007)	*h* = −10→11
*T*_min_ = 0.567, *T*_max_ = 0.638	*k* = −8→11
6139 measured reflections	*l* = −18→14

### Refinement

**Table:**

Refinement on *F*^2^	Secondary atom site location: difference Fourier map
Least-squares matrix: full	Hydrogen site location: inferred from neighbouring sites
*R*[*F*^2^ > 2σ(*F*^2^)] = 0.026	H-atom parameters constrained
*wR*(*F*^2^) = 0.065	*w* = 1/[σ^2^(*F*_o_^2^) + (0.0284*P*)^2^ + 2.1868*P*] where *P* = (*F*_o_^2^ + 2*F*_c_^2^)/3
*S* = 1.14	(Δ/σ)_max_ = 0.001
708 reflections	Δρ_max_ = 0.68 e Å^−^^3^
75 parameters	Δρ_min_ = −0.39 e Å^−^^3^
0 restraints	Absolute structure: Flack (1983), 235 Friedel pairs
Primary atom site location: structure-invariant direct methods	Flack parameter: 0.01 (3)

### Special details

**Table:**

Geometry. All e.s.d.'s (except the e.s.d. in the dihedral angle between two l.s. planes) are estimated using the full covariance matrix. The cell e.s.d.'s are taken into account individually in the estimation of e.s.d.'s in distances, angles and torsion angles; correlations between e.s.d.'s in cell parameters are only used when they are defined by crystal symmetry. An approximate (isotropic) treatment of cell e.s.d.'s is used for estimating e.s.d.'s involving l.s. planes.
Refinement. Refinement of *F*^2^ against ALL reflections. The weighted *R*-factor *wR* and goodness of fit *S* are based on *F*^2^, conventional *R*-factors *R* are based on *F*, with *F* set to zero for negative *F*^2^. The threshold expression of *F*^2^ > σ(*F*^2^) is used only for calculating *R*-factors(gt) *etc*. and is not relevant to the choice of reflections for refinement. *R*-factors based on *F*^2^ are statistically about twice as large as those based on *F*, and *R*– factors based on ALL data will be even larger.

### Fractional atomic coordinates and isotropic or equivalent isotropic displacement parameters (Å^2^)

**Table:**

	*x*	*y*	*z*	*U*_iso_*/*U*_eq_	
Ni1	0.55533 (4)	0.44467 (4)	0.0833	0.0098 (2)	
P2	0.38859 (12)	0.21675 (12)	0.24795 (7)	0.0093 (3)	
O5	0.4156 (3)	0.2355 (3)	0.34570 (16)	0.0108 (6)	
O4	0.2137 (3)	0.1899 (4)	0.23106 (18)	0.0129 (7)	
O3	0.4865 (4)	0.1970 (4)	0.05090 (19)	0.0214 (7)	
O2	0.5200 (4)	0.3782 (3)	0.21028 (17)	0.0142 (7)	
O1	0.3853 (4)	0.0644 (4)	0.21452 (17)	0.0151 (7)	
O6	0.2044 (10)	0.1022 (5)	−0.0833	0.079 (2)	
B	0.3037 (8)	0.1518 (4)	0.4167	0.0090 (13)	
Li	0.466 (3)	0.2331 (13)	−0.0833	0.080 (5)	
H3A	0.5738	0.2196	0.0284	0.096*	
H6	0.1509	0.0382	−0.1223	0.096*	
H3B	0.4428	0.1571	0.0973	0.096*	

### Atomic displacement parameters (Å^2^)

**Table:**

	*U*^11^	*U*^22^	*U*^33^	*U*^12^	*U*^13^	*U*^23^
Ni1	0.0098 (3)	0.0098 (3)	0.0100 (3)	0.0050 (3)	0.0013 (3)	0.0013 (3)
P2	0.0097 (5)	0.0097 (5)	0.0086 (5)	0.0050 (4)	0.0015 (4)	0.0015 (4)
O5	0.0095 (14)	0.0126 (16)	0.0089 (13)	0.0044 (12)	0.0014 (11)	0.0016 (11)
O4	0.0131 (16)	0.0132 (15)	0.0157 (16)	0.0091 (13)	−0.0022 (12)	−0.0030 (12)
O3	0.0277 (18)	0.0162 (18)	0.0231 (17)	0.0130 (14)	0.0121 (14)	0.0049 (14)
O2	0.0143 (16)	0.0134 (14)	0.0101 (13)	0.0032 (13)	0.0021 (12)	0.0040 (11)
O1	0.0211 (17)	0.0159 (16)	0.0147 (14)	0.0140 (14)	0.0008 (14)	−0.0017 (12)
O6	0.083 (6)	0.061 (3)	0.100 (6)	0.042 (3)	0.000	−0.024 (4)
B	0.012 (3)	0.009 (2)	0.007 (3)	0.0059 (15)	0.000	0.001 (2)
Li	0.094 (15)	0.089 (10)	0.058 (10)	0.047 (8)	0.000	0.011 (10)

### Geometric parameters (Å, °)

**Table:**

Ni1—O1^i^	2.048 (3)	O4—B^i^	1.471 (5)
Ni1—O1^ii^	2.048 (3)	O3—Li	2.164 (4)
Ni1—O2^iii^	2.070 (3)	O2—Li^iv^	2.113 (13)
Ni1—O2	2.070 (3)	O1—Ni1^v^	2.048 (3)
Ni1—O3	2.130 (3)	O6—Li	2.12 (2)
Ni1—O3^iii^	2.130 (3)	B—O5^vi^	1.461 (5)
Ni1—Li	3.137 (5)	B—O4^vii^	1.471 (5)
Ni1—Li^iv^	3.137 (5)	B—O4^v^	1.471 (5)
P2—O1	1.503 (3)	Li—O2^iii^	2.113 (13)
P2—O2	1.510 (3)	Li—O2^viii^	2.113 (13)
P2—O4	1.546 (3)	Li—O3^ix^	2.164 (4)
P2—O5	1.556 (3)	Li—Ni1^viii^	3.137 (5)
O5—B	1.461 (5)		
			
O1^i^—Ni1—O1^ii^	92.58 (18)	B—O5—P2	131.6 (3)
O1^i^—Ni1—O2^iii^	88.53 (11)	B^i^—O4—P2	127.8 (3)
O1^ii^—Ni1—O2^iii^	101.19 (12)	Ni1—O3—Li	93.87 (18)
O1^i^—Ni1—O2	101.19 (12)	P2—O2—Ni1	127.36 (17)
O1^ii^—Ni1—O2	88.53 (11)	P2—O2—Li^iv^	129.2 (4)
O2^iii^—Ni1—O2	166.00 (17)	Ni1—O2—Li^iv^	97.1 (2)
O1^i^—Ni1—O3	86.52 (13)	P2—O1—Ni1^v^	140.72 (18)
O1^ii^—Ni1—O3	177.55 (12)	O5^vi^—B—O5	103.4 (4)
O2^iii^—Ni1—O3	81.08 (11)	O5^vi^—B—O4^vii^	111.43 (15)
O2—Ni1—O3	89.40 (11)	O5—B—O4^vii^	114.16 (15)
O1^i^—Ni1—O3^iii^	177.55 (12)	O5^vi^—B—O4^v^	114.16 (15)
O1^ii^—Ni1—O3^iii^	86.52 (13)	O5—B—O4^v^	111.43 (16)
O2^iii^—Ni1—O3^iii^	89.40 (11)	O4^vii^—B—O4^v^	102.6 (4)
O2—Ni1—O3^iii^	81.08 (11)	O2^iii^—Li—O2^viii^	106.9 (9)
O3—Ni1—O3^iii^	94.47 (19)	O2^iii^—Li—O6	126.5 (5)
O1^i^—Ni1—Li	72.0 (4)	O2^viii^—Li—O6	126.5 (5)
O1^ii^—Ni1—Li	138.23 (8)	O2^iii^—Li—O3	79.3 (3)
O2^iii^—Ni1—Li	41.9 (3)	O2^viii^—Li—O3	95.5 (4)
O2—Ni1—Li	131.83 (17)	O6—Li—O3	94.3 (6)
O3—Ni1—Li	43.50 (9)	O2^iii^—Li—O3^ix^	95.5 (4)
O3^iii^—Ni1—Li	107.3 (4)	O2^viii^—Li—O3^ix^	79.3 (3)
O1^i^—Ni1—Li^iv^	138.23 (8)	O6—Li—O3^ix^	94.3 (6)
O1^ii^—Ni1—Li^iv^	72.0 (4)	O3—Li—O3^ix^	171.3 (11)
O2^iii^—Ni1—Li^iv^	131.83 (17)	O2^iii^—Li—Ni1	40.91 (9)
O2—Ni1—Li^iv^	41.9 (3)	O2^viii^—Li—Ni1	118.8 (6)
O3—Ni1—Li^iv^	107.3 (4)	O6—Li—Ni1	103.3 (4)
O3^iii^—Ni1—Li^iv^	43.50 (9)	O3—Li—Ni1	42.64 (13)
Li—Ni1—Li^iv^	143.2 (5)	O3^ix^—Li—Ni1	134.5 (3)
O1—P2—O2	115.38 (17)	O2^iii^—Li—Ni1^viii^	118.8 (6)
O1—P2—O4	105.45 (17)	O2^viii^—Li—Ni1^viii^	40.91 (9)
O2—P2—O4	111.07 (18)	O6—Li—Ni1^viii^	103.3 (4)
O1—P2—O5	112.24 (16)	O3—Li—Ni1^viii^	134.5 (4)
O2—P2—O5	105.75 (16)	O3^ix^—Li—Ni1^viii^	42.64 (13)
O4—P2—O5	106.70 (15)	Ni1—Li—Ni1^viii^	153.4 (7)

Symmetry codes: (i) *x*−*y*, *x*, *z*−1/6; (ii) −*x*+1, −*x*+*y*+1, −*z*+1/3; (iii) −*y*+1, −*x*+1, −*z*+1/6; (iv) −*x*+*y*+1, −*x*+1, *z*+1/3; (v) *y*, −*x*+*y*, *z*+1/6; (vi) *x*, *x*−*y*, −*z*+5/6; (vii) *y*, *x*, −*z*+2/3; (viii) −*y*+1, *x*−*y*, *z*−1/3; (ix) *x*, *x*−*y*, −*z*−1/6.

### Hydrogen-bond geometry (Å, °)

**Table:**

*D*—H···*A*	*D*—H	H···*A*	*D*···*A*	*D*—H···*A*
O3—H3A···O5^viii^	0.81	2.01	2.746 (4)	151
O3—H3A···O2^viii^	0.81	2.60	3.165 (4)	128
O6—H6···O4^x^	0.83	2.52	3.331 (4)	167
O6—H6···O1^x^	0.83	2.66	3.092 (4)	114
O3—H3B···O1	0.83	2.00	2.810 (4)	167
O3—H3B···O2	0.83	2.54	2.955 (4)	112

Symmetry codes: (viii) −*y*+1, *x*−*y*, *z*−1/3; (x) *x*−*y*, −*y*, −*z*.

## References

[bb1] Boy, I. & Kniep, R. J. (2001). *Z. Kristallogr. New Cryst. Struct.* **216**, 9–10.

[bb2] Bruker (2007). *APEX2*, *SAINT* and *SADABS* Bruker AXS Inc., Madison, Wisconsin, USA.

[bb3] Ewald, B., Huang, Y.-X. & Kniep, R. (2007). *Z. Anorg. Allg. Chem* **633**, 1517–1540.

[bb4] Flack, H. D. (1983). *Acta Cryst.* A**39**, 876–881.

[bb5] Kniep, R., Engelhardt, H. & Hauf, C. (1998). *Chem. Mater.* **10**, 2930–2934.

[bb6] Lin, J.-R., Huang, Y.-X., Wu, Y.-H. & Zhou, Y. (2008). *Acta Cryst.* E**64**, i39–i40. 10.1107/S160053680801516X PMC2961425 21202441

[bb7] Sheldrick, G. M. (2008). *Acta Cryst.* A**64**, 112–122. 10.1107/S0108767307043930 18156677

